# Hamman Syndrome: A Case Report of Interlobar Pneumothorax, Pneumopericardium, and Pneumorrhachis

**DOI:** 10.7759/cureus.72825

**Published:** 2024-11-01

**Authors:** Fábio Ferreira, Miguel Castro

**Affiliations:** 1 Department of Radiology, Unidade Local de Saúde de São João, Porto, PRT

**Keywords:** hamman syndrome, interlobar pneumothorax, pneumopericardium, pneumorrhachis, spontaneous pneumomediastinum, subcutaneous emphysema

## Abstract

Hamman syndrome is a rare and benign condition characterized by the presence of air in the mediastinum without an external cause. It most commonly occurs in young adults and is often triggered by activities that increase intrathoracic pressure, such as coughing, vomiting, or asthma exacerbations.

We report the case of an 18-year-old male patient with a history of asthma who presented with shortness of breath, chest pain, and subcutaneous emphysema. Imaging evaluation revealed pneumomediastinum, interlobar pneumothorax, pneumopericardium, pneumorrhachis, and subcutaneous emphysema. The patient was treated with supplemental oxygen and analgesia. The symptoms gradually improved, and follow-up imaging confirmed the resolution of pneumomediastinum within four days.

Hamman syndrome is commonly misdiagnosed due to its non-specific clinical presentation. Imaging evaluation is crucial for diagnosis and for differentiating it from other serious conditions. This case report illustrates a rare combination of uncommon imaging findings in Hamman syndrome.

## Introduction

Hamman syndrome, also known as spontaneous pneumomediastinum, is an uncommon and benign condition characterized by the presence of air within the mediastinum without any external or traumatic cause. The underlying pathophysiology presupposes that rupture of alveoli due to increased intra-alveolar pressure allows the air to dissect along the bronchovascular sheaths into the mediastinum, a process known as the Macklin effect [1]. Common triggers for this condition include intense coughing, asthma exacerbations, vomiting, and barotrauma [2].

Hamman syndrome most often occurs in young adults, particularly male patients, typically between the second and fourth decades of life [3]. There is also a notable prevalence in peripartum and postpartum women [4]. Patients usually report non-specific symptoms such as chest pain, shortness of breath, and coughing. Subcutaneous emphysema is the most frequent finding on physical examination [5].

This syndrome is often misdiagnosed or overlooked during initial assessments, due to its spontaneous and non-specific presentation. Therefore, imaging studies are crucial for diagnosis and management. Chest radiographs and computed tomography (CT) scans are the primary imaging modalities used to detect air in the mediastinum, monitor the resolution of pneumomediastinum, and distinguish it from more severe conditions, such as Boerhaave syndrome and tension pneumothorax [6,7].

## Case presentation

An 18-year-old male patient, a non-smoker, with a history of controlled asthma without regular medication, presented to the emergency department due to an acute episode of shortness of breath, coughing, and chest pain. Despite initial symptomatic relief with salbutamol, the patient developed worsening chest pain and noticed swelling in the anterior cervical region. There was no history of trauma or recent sports activity.

Upon evaluation in the emergency department, his vital signs were within normal parameters, with an oxygen saturation of 98%, and did not show signs of respiratory distress. Cardiopulmonary auscultation was unremarkable. A mild subcutaneous emphysema in the anterior cervical region was noted. Laboratory workup did not reveal any abnormalities, and an electrocardiogram showed a normal sinus rhythm without any pathological findings.

**Figure 1:**
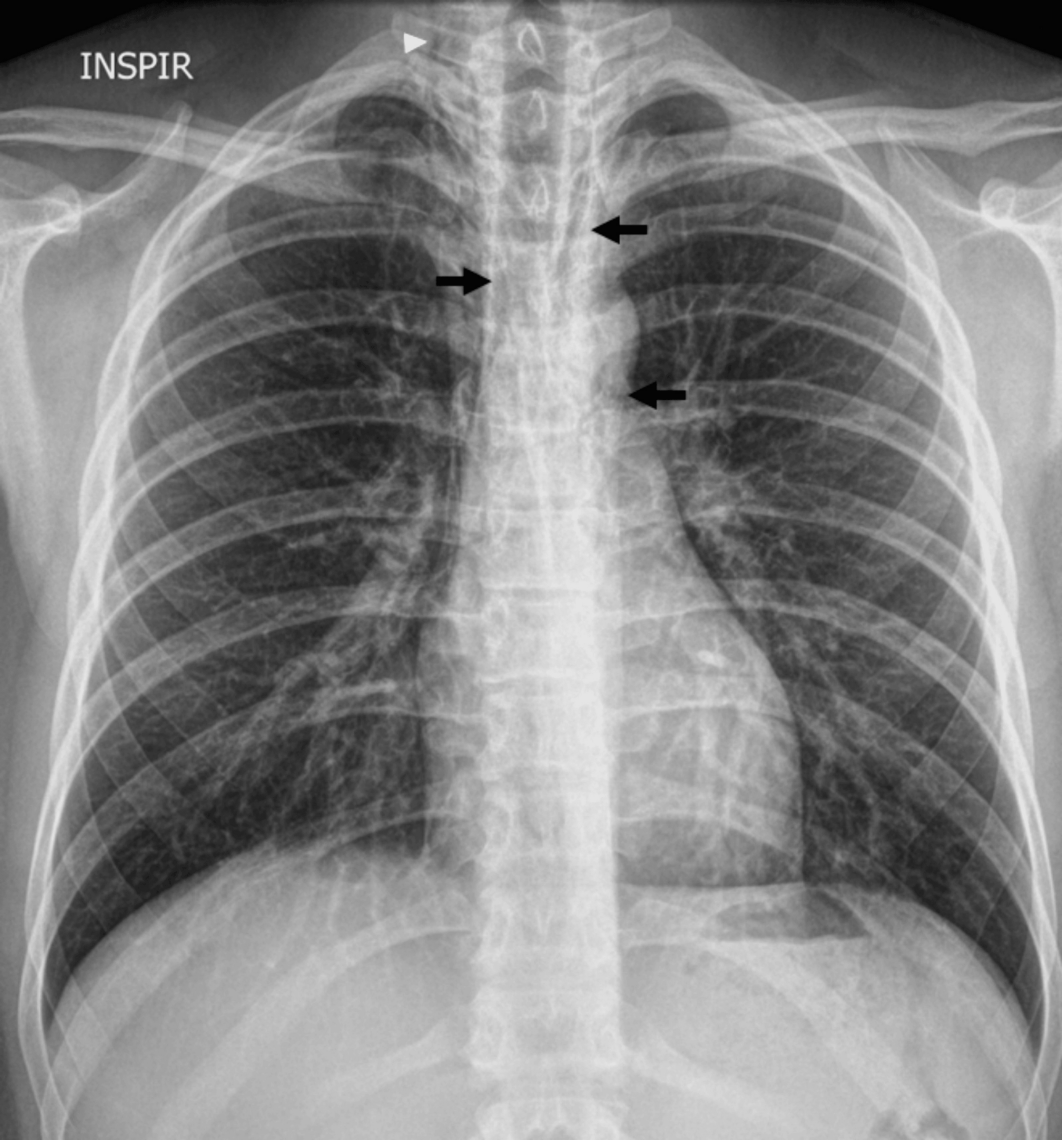
Posteroanterior chest radiograph shows linear lucencies in the mediastinum (arrows), suggesting pneumomediastinum. Additionally, air is noted in the soft tissues of the cervical region (arrowhead).

The initial imaging assessment was made with a posteroanterior chest radiograph that showed air in the mediastinum, particularly evident lateral to the trachea and in the aortic knob, consistent with pneumomediastinum. The air was noted to extend superiorly into the soft tissues of the cervical region (Figure 1).

**Figure 2:**
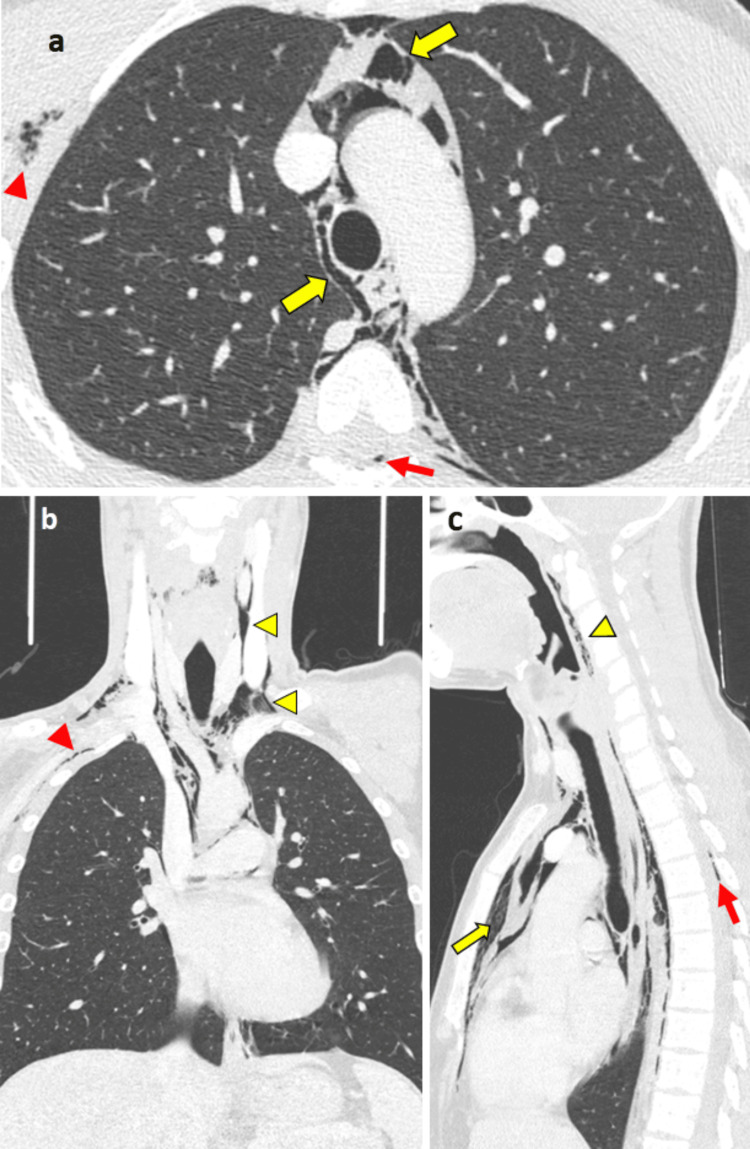
Axial (a), coronal (b), and sagittal (c) CT scan (lung window) reveals the presence of pneumomediastinum (yellow arrows) and pneumorrhachis (red arrows). The air extends into the soft tissues of the neck (yellow arrowheads) and the right chest wall (red arrowheads). CT: computed tomography

**Figure 3:**
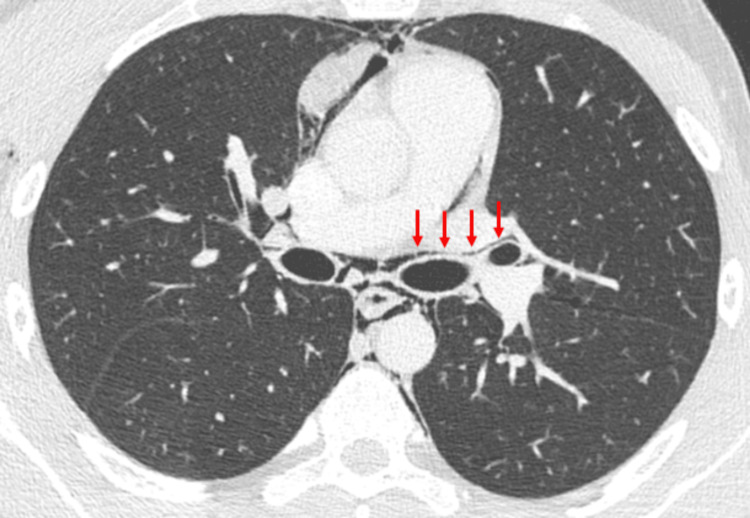
Axial CT scan (lung window) reveals linear air bands (arrows) along the left bronchovascular sheath, known as the Macklin effect. CT: computed tomography

To further assess the extent of pneumomediastinum and exclude possible causes of pneumomediastinum, a non-contrast chest CT scan was performed. The CT scan confirmed the presence of pneumomediastinum, as well as subcutaneous emphysema in the neck and right chest wall (Figure 2). Linear air bands were visible along the left bronchovascular sheath (Figure 3).

**Figure 4:**
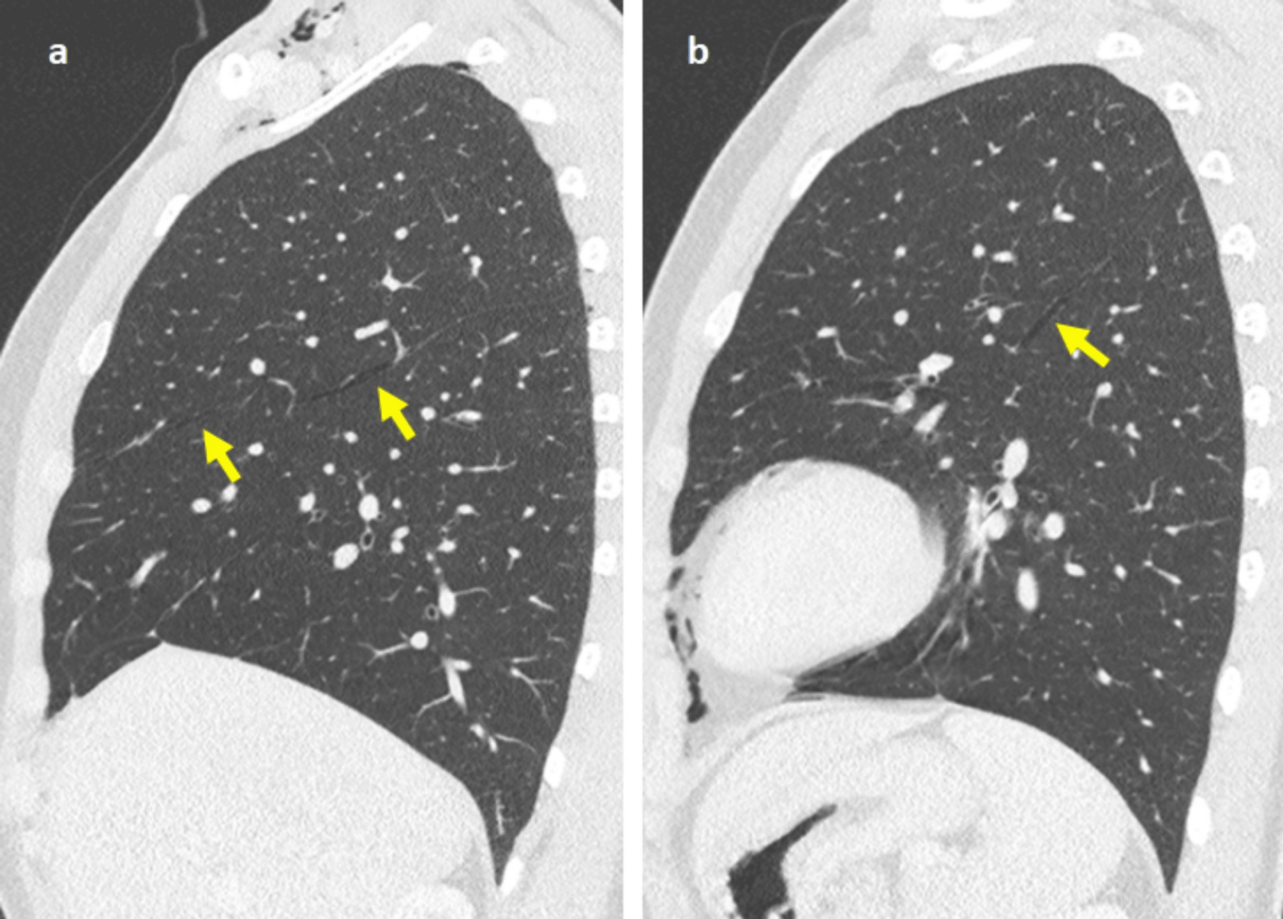
Sagittal CT scan (lung window) shows pneumothorax (arrows) in the right oblique and horizontal fissures (a), as well as in the left oblique fissure (b). CT: computed tomography

**Figure 5:**
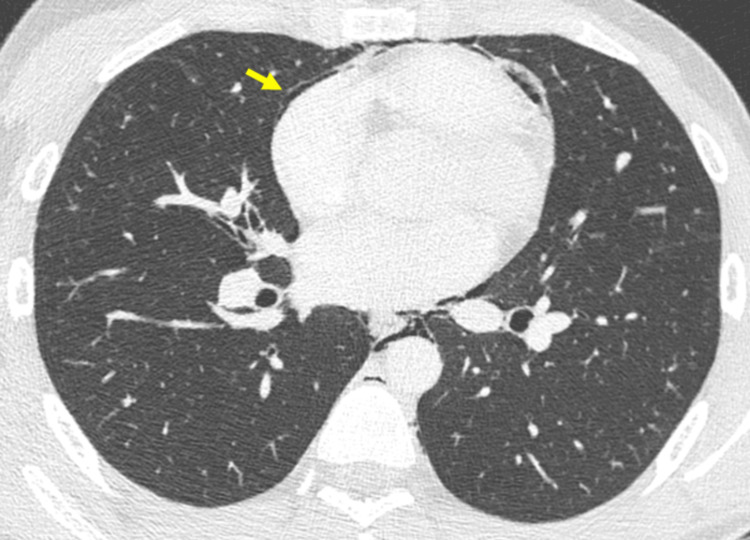
Axial CT scan (lung window) demonstrates a small volume pneumopericardium (arrow). CT: computed tomography

A small bilateral pneumothorax was present, with air along the pulmonary fissures, an uncommon manifestation known as interlobar pneumothorax (Figure 4). A small amount of air was also observed in the spinal canal (pneumorrhachis) and the pericardial cavity (pneumopericardium) (Figure 2 and Figure 5).

There were no visible pathological findings in the lung parenchyma, namely nodules, consolidations, cavitations, or bullous emphysema. Furthermore, there was no evidence of esophageal or tracheobronchial perforation.

**Figure 6:**
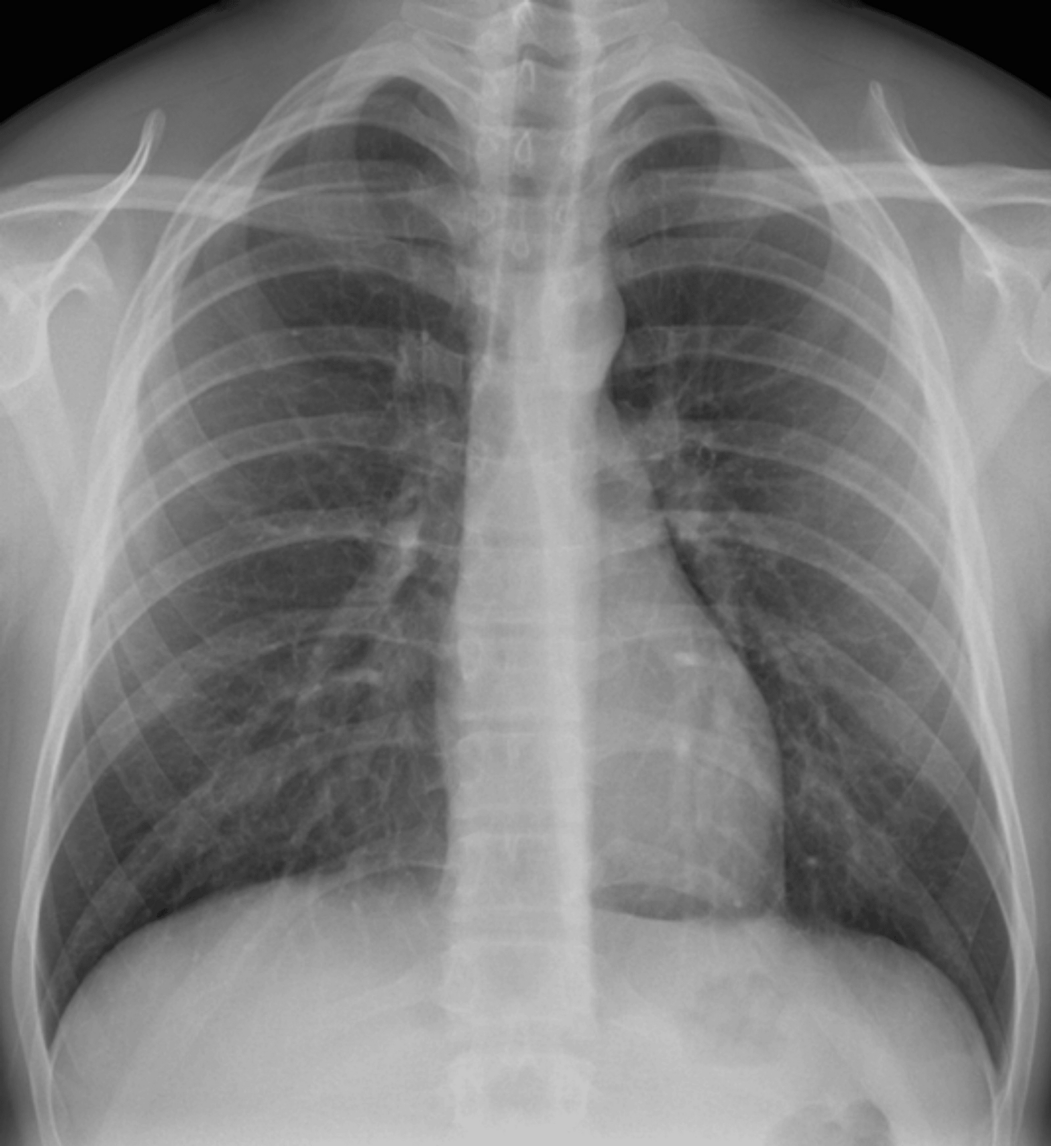
Posteroanterior chest radiograph obtained four days after symptom onset confirming the resolution of the initial imaging abnormalities.

The clinical and imagological findings were consistent with Hamman syndrome. A conservative approach was adopted, including monitoring, pain control, and supplemental oxygen. The patient's symptoms gradually improved, and follow-up imaging four days after the onset of the symptoms demonstrated resolution of the pneumomediastinum (Figure 6).

## Discussion

Hamman syndrome refers to the spontaneous development of pneumomediastinum, a phenomenon wherein air accumulates in the mediastinal space. The underlying mechanism involves the rupture of alveoli secondary to increased intra-alveolar pressure, a process referred to as the Macklin effect. This rupture allows air to escape into the pulmonary interstitium and migrate along the peribronchovascular sheaths toward the mediastinum [1]. The air can also spread to the pericardium, neck, subcutaneous tissue, and peritoneum. Trapped air between the parietal and visceral pleura results in the development of pneumothorax [4].

Spontaneous pneumomediastinum usually occurs after activities that increase intrathoracic pressure, such as severe coughing, vomiting, and physical exercise. In individuals with predisposing factors such as asthma, the sudden increase in airway pressure during acute exacerbations can precipitate this syndrome [2]. In the case presented, the patient's asthma likely contributed to the development of pneumomediastinum.

The most frequent clinical manifestations of Hamman syndrome are chest pain, shortness of breath, and subcutaneous emphysema. The pain is often retrosternal and can radiate to the neck or back. The presence of subcutaneous emphysema is often the clinical clue that suggests the diagnosis. Additional less frequent symptoms include odynophagia, cough, dysphonia, and dysphagia [7]. In this case, the patient's symptoms of chest pain and shortness of breath were consistent with typical symptoms. The Hamman's sign, a crunching sound over the mediastinum synchronous with the cardiac cycle, is pathognomonic of pneumomediastinum. However, it is only present in a minority of patients [5].

Since the symptoms and physical examination are non-specific, imaging evaluation is essential for diagnosing Hamman syndrome. A chest radiograph is frequently the initial imaging modality to evaluate patients with chest pain. The key radiographic finding in pneumomediastinum is the presence of linear or curvilinear lucencies surrounding the mediastinal structures [7].

CT is the imaging modality of choice for pneumomediastinum diagnosis, providing superior resolution and detail compared to plain radiograph, which underestimates the volume of pneumomediastinum [8]. The CT scan in this patient clearly demonstrated free air in the mediastinum and subcutaneous emphysema.

A particularly rare aspect of this case was the combination of interlobar pneumothorax, pneumorrhachis, and pneumopericardium, all of which are uncommon in Hamman syndrome. Pleural air was observed in the space between the pulmonary lobes, referred to as interlobar pneumothorax. This presentation form of pneumothorax is rare, but its causes and management are similar to those of a typical pneumothorax [9].

Pneumopericardium refers to the accumulation of air in the pericardial cavity, most commonly associated with chest trauma in adults. In some cases, patients may develop cardiac tamponade, a condition with a high mortality rate. Pneumopericardium can also be a complication of pneumothorax or pneumomediastinum [10]. In the context of Hamman syndrome, pneumopericardium is usually benign and self-limiting [11].

Pneumorrhachis is the presence of gas within the spinal canal, and it is usually associated with trauma, spinal surgery, and epidural anesthesia. However, it is also a rare finding described in Hamman syndrome cases. In cases of pneumomediastinum, the air can communicate between the posterior mediastinum and the epidural space, due to the lack of a fascial barrier. In most cases, pneumorrhachis is an asymptomatic and self-limiting condition, whose treatment is conservative [12,13].

The differential diagnosis of Hamman syndrome includes life-threatening pathologies such as mediastinitis, Boerhaave syndrome, and tension pneumothorax. Each of these conditions has distinct radiological features that help distinguish them from Hamman syndrome. Mediastinitis mostly occurs after cardiovascular or cardiothoracic surgical procedures or as a result of the spread of oropharyngeal infections. In these cases, there are mediastinal inflammation signs, such as mediastinal fat stranding, edema, and abscesses. Boerhaave syndrome corresponds to an esophageal rupture, usually following severe vomiting. In addition to pneumomediastinum, focal esophageal wall thickening and periesophageal fluid collections are frequently detected [6]. In tension pneumothorax, air accumulates in the pleural space and compresses the lung and mediastinum, with consequent hemodynamic compromise. Radiological findings include collapsed lung on the affected side, mediastinal shift to the contralateral side, and flattened diaphragm [14].

The management of Hamman syndrome is primarily conservative. In most cases, patients require only monitoring, analgesia for chest pain, and supplemental oxygen, which promote rapid reabsorption of air from the mediastinum [5]. Usually, patients are admitted to the hospital for two to five days for clinical vigilance [7]. Pneumomediastinum disappears within a few days to weeks without any long-term sequelae. Serial imaging with chest radiographs can be used to confirm the resolution of pneumomediastinum [5].

In the reported case, the patient demonstrated gradual symptom improvement, and follow-up imaging confirmed the resolution of pneumomediastinum within four days, illustrating the generally favorable prognosis associated with Hamman syndrome.

## Conclusions

Hamman syndrome typically presents in young adults with chest pain and shortness of breath. Imaging evaluation, especially with CT, is crucial not only for diagnosing spontaneous pneumomediastinum but also for ruling out other life-threatening conditions, such as tension pneumothorax or mediastinitis. Early recognition and differentiation from other serious conditions are essential to avoid unnecessary interventions and ensure optimal patient care. Conservative management, including monitoring, symptomatic relief, and supplemental oxygen, is the gold standard treatment. This case report of Hamman syndrome illustrates a rare combination of uncommon imaging findings, namely interlobar pneumothorax, pneumopericardium, and pneumorrhachis.
